# Prevalence and characteristics of dengue virus co-infection in patients and mosquitoes collected from patients’ houses

**DOI:** 10.1371/journal.pone.0314553

**Published:** 2025-03-27

**Authors:** Supranee Phanthanawiboon, Tipaya Ekalaksananan, Jureeporn Chuerduangphui, Apiporn Thinkhamrop Suwannatrai, Sirinart Aromseree, Neal Alexander, Hans J. Overgaard, Panwad Thongchai, Ati Burassakarn, Chamsai Pientong

**Affiliations:** 1 Department of Microbiology, Faculty of Medicine, Khon Kaen University, Khon Kaen, Thailand; 2 HPV & EBV and Carcinogenesis Research Group, Khon Kaen University, Khon Kaen, Thailand; 3 Department of Parasitology, Faculty of Medicine, Khon Kaen University, Khon Kaen, Thailand; 4 MRC International Statistics and Epidemiology Group, London School of Hygiene and Tropical Medicine, London, United Kingdom; 5 Faculty of Science and Technology, Norwegian University of Life Sciences, Ǻs, Norway; Institute of Tropical Medicine: Instituut voor Tropische Geneeskunde, BELGIUM

## Abstract

Co-infection with multiple DENV serotypes can affect human immune status and complicate the clinical presentation and management of dengue patients, so understanding the prevalence and dynamics of co-infection is important for effective dengue control. We aimed to identify and characterize DENV co-infection patterns in field-caught mosquitoes and dengue patients. This study was conducted in northeastern Thailand between June 2016 to August 2019. Female *Aedes* mosquitos collected from and around dengue patient’s houses were analyzed for DENV infection and presence of serotypes using RT-PCR. DENV serotyping was successful in 154 (39.49%) of human and 165 (14.26%) of mosquito samples. Prevalence of DENV co-infection in patients and mosquitoes was 22.73% (35 cases) and 28.48% (47 samples), respectively. Co-infection with multiple serotypes were double (human 88.57%, mosquito 89.36%), triple (human 5.72%, mosquito 10.64%) and quadruple (human 5.72%, mosquito 0%) infections. Concurrent infection was different between hosts and concurrence patterns of DENV serotype in each host mostly composed of the predominant serotype of the detected year. This is the first report that show DENV co-infection patterns in field-caught mosquito and in dengue fever patients with combinations of triple and quadruple serotypes in Thailand. These finding are potentially useful for understanding shifts in serotypes, concurrent DENV infection patterns, vaccine development, and further research on the ability of vectors to transmit multiple serotypes.

## Introduction

Dengue virus (DENV), the fastest spreading mosquito-borne virus causing a variety of symptoms from mild to severe in humans, which can lead to death. Dengue has been classified by the World Health Organization (WHO) into dengue fever (DF), dengue hemorrhagic fever (DHF) and dengue shock syndrome (DSS) [1]. In 2009, WHO changed the grading criteria to dengue with or without warning signs [2,3]. DENV consists of 4 serotypes; DENV1, DENV2, DENV3 and DENV4, which are serologically and genetically distinct [4]. Severity of the disease depend on many factors including the serotype of the virus which is involved in viremic status, sequence of viral strain in infection and preexisting immunity of a host [5–8]. *Aedes aegypti* is the main DENV vector but other mosquito species have been reported to be competent [9]. Due to urbanization, globalization and the lack of effective virus and vector control interventions, the expansion of the virus and transmission of new serotypes globally has made dengue an important public health problem especially in tropical and subtropical countries. In hyperendemic countries, more than one serotype can co-circulate [10,11], which can lead to severe infection due to the ability of preexisting immunity to enhance distinct serotypes to infect host cells. Preexisting antibodies may determine the host susceptibility to DENV serotypes resulting in dominant serotype replacement each year [12,13].

The circulation of multiple DENV serotypes in a hyperendemic region increases the chances of co-infection or concurrent infection in an individual. However, it is still not clear whether this is a result of bites from mosquitoes carrying different serotypes at subsequent bites or from a bite from a single mosquito carrying multiple serotypes [14]. Recent reports from many countries have indicated human co-infection with multiple DENV serotypes, mostly during outbreaks [10,17]. Immunity from infection of DENV related to the clinical outcome could also play a role in shaping the evolutionary and epidemiological dynamics of DENV [5,15]. However, less is known in case of co-infection. Some studies have shown the association of co-infection with increased risk of severe dengue based on clinical and laboratory results [16], while others have shown no significant change in clinical and laboratory results compared to mono-infection cases [17]. Most studies showed co-infection with two DENV serotypes.

Recently, our group reported prevalence and molecular studies of dengue virus in human and field-caught mosquitoes in four provinces of northeastern Thailand [18,19]. The objective of this study was to characterize co-infection of dengue in human and mosquito samples. To understand co-infection characteristics, we showed the prevalence of DENV co-infection patterns and characteristics of dynamic change of DENV either in mono-infection and co-infection during 2016-2019. Moreover, the laboratory profiles of dengue patients were analyzed. These results will facilitate the understanding of DENV serotype distribution pattern between human and mosquito and dynamic change of DENV co-infection in mosquito and patients in hyperendemic countries.

## Materials and methods

### Ethics

This study was conducted with the approval of Khon Kaen University Ethics Committee for Human Research (KKUEC, no. HE611454), the London School of Hygiene and Tropical Medicine Ethical Committee, UK (no. 10534), and the Regional Committee for Medical and Health Research Ethics, Section B, South-East Norway (no.2016/357). These ethical approvals are in accordance with Helsinki Declaration of 1975. The experiments were performed in accordance with relevant guidelines and regulations. All patients in this study provided informed consent before blood was taken.

### Sample collection

All plasma and mosquito samples were obtained from a hospital-based case-control study which conducted in 11 hospitals in four provinces including Khon Kaen, Roi Et, Kalasin, Chaiyaphum and Maha Sarakham in northeastern Thailand from June 2016 to August 2019. The method of collection was described in a previous study [20]. In brief, acute febrile illness patients were recruited from participating hospitals. Inclusion criteria were patients being five years or older with fever (body temperature >  38°C). Exclusion criteria were i) residence outside the hospitals’ catchment areas, ii) travel away from the residential area during the previous seven days, and iii) inability to sign the consent form due to disease or other reasons. After signing the consent form, DENV screening test was performed using the SD BIOLINE Dengue Duo kit that included NS1, IgM and IgG (Standard Diagnostics, Suwon, Korea), according to the manufacturer’s instructions before hospitalization where the blood was collected for laboratory. Primary and secondary infection status was identified based on the presence of IgM and IgG. Primary infection was classified as the presence of IgM or NS1 or NS1 with IgM. Secondary infection was classified as the presence of IgG with NS1 or IgG with IgM. Demographic and laboratory data including sex, age and complete blood count profiles were collected from the hospital. Adult mosquitoes were collected by Prokopack aspirators for 15 minutes indoors and outdoors from the patient’s house and four additional neighboring houses within 24 h of the consent being signed.

### Dengue detection in mosquito and human blood samples

Laboratory-confirmed DENV detection and serotyping were carried out in the laboratory at Khon Kaen University, Khon Kaen province, Thailand. Adult mosquitoes were identified to species by expert staff from the office of Disease Prevention and Control, Ministry of Public Health in the provinces of sample collection. Only adult female *Aedes* were used in this study. Mosquitoes were cut to separate head/thoraxes from abdomens, then kept individually at -20°C. Before homogenization, pools of up to 10 abdomens were made. When a pool was found to be positive, the corresponding heads/thoraxes were then analyzed separately. Negative abdomen pools did not lead to further analysis. Sample tubes were dipped into liquid nitrogen for 10 seconds before the sample was mashed by sterile pestle. Ice-cooled L-15 medium (Hyclone Laboratories Inc, USA) 500 μL and 300 μL was added to the abdomen pool and individual head/thoraxes sample tube respectively. Clear supernatant was separated from cell lysate by 12,500 rpm centrifugation before subjected for RNA extraction by using QIAamp Viral RNA Mini kits (Qiagen, Germany). QIAamp viral RNA Mini kits (Qiagen, Germany) was also used for RNA extraction from human blood sample following the protocol. Dengue detection and serotyping in human samples were performed using the primer set from Shu et al. as presented in Table 1 [21]. The real-time PCR reaction was performed with total volume 10 µl on an Applied Biosystems® 7500 Real-Time PCR machine. Each reaction contained 1x SsoAdvanced™ Universal SYBR® Green Supermix (Bio-Rad, Hercules, CA, USA), 0.3 µ M of each forward and reverse primer, and 20 ng of cDNA template. The thermal cycling conditions were 3 minutes of denaturation at 94°C, followed by 45 cycles of denaturation at 94°C for 10 seconds and annealing/extension at 55°C for 90 seconds. For dengue virus detection and serotyping in mosquito samples, we used the protocol by Lanciotti et al. with minor adaptation to the real-time PCR platform, as described in a previous study [19], with primer details shown in Table 2 [22]. Briefly, viral RNA from pooled samples was reverse-transcribed into cDNA using the specific D2-Rv primer with the SuperScript III First-Strand Synthesis System (Invitrogen, USA), according to the manufacturer’s instructions, and stored at − 20°C until use. The first round of PCR aimed to screen for DENV infection in pooled mosquito samples using SYBR Green-based real-time PCR with the D1-Fw and D2-Rv primers from Lanciotti et al. Positive result showed Tm around 85.6°C. The product from real-time PCR at 511 bp was confirmed by gel electrophoresis (S1A Fig). All individual mosquito specimens corresponding to each dengue-positive pool were subjected to the second round of PCR for DENV serotyping, also using the primer set of Lanciotti et al. The serotyping PCR reaction included pre-denaturation at 95°C for 2 minutes, followed by 35 cycles of 95°C for 15 seconds, 55°C for 30 seconds, and a final extension at 72°C for 40 seconds. PCR amplification was carried out on an Applied Biosystems® 7500 Real-Time PCR machine (Applied Biosystems, CA, USA). The Tm of DENV serotype 1-4 showed ranged from 84.3°C to 85.8°C respectively and all reactions were verified using 2% gel electrophoresis (S1A Fig), with some positive samples selected for sequencing for confirmation.

**Table 1:** Primers for DENV detection and serotyping in human samples by SYBR green real time PCR describe by Shu et al., 2003 [21].

Primer name	Primer sequence
DN-Fw	CAA TAT GCT GAA ACG CGA GAG AAA
DN-Rv	CCC CAT CTA TTC AGA ATC CCT GCT
D1-Rv	CGC TCC ATA CAT CTT GAA TGA G
D2-Rv	AAG ACA TTG ATG GCT TTT GA
D3-Rv	AAG ACG TAA ATA GCC CCC GAC
D4-Rv	AGG ACT CGC AAA AAC GTG ATG AAT

**Table 2:** Primers for DENV detection and serotyping in mosquito samples by SYBR green real time PCR describe by Lanciotti et al., 1992 [22].

Primer name	Primer sequence (5’-3’)
Dl-Fw	TCA ATA TGC TGA AAC GCG CGA GAA ACC G
D2-Rv	TTG CAC CAA CAG TCA ATG TCT TCA GGT TC
TS1-Rv	CGT CTC AGT GAT CCG GGG G
TS2-Rv	CGC CAC AAG GGC CAT GAA CAG
TS3-Rv	TAA CAT CAT CAT GAG ACA GAG C
TS4-Rv	CTC TGT TGT CTT AAA CAA GAG A

### Analysis

The location of households with DENV mono- and co-infection in human and mosquito samples during 2016-2019 were georeferenced using global positioning devices to identify infection areas. The administrative boundaries were obtained from the Global Administrative Areas Database (GADM), available at https://www.gadm.org. The maps were generated using Quantum GIS (QGIS) version 3.28.0, an open-source software available at https://www.qgis.org/.

Demographic data were statistically analyzed using Student’s t-test and Fisher’s exact test.

## Results

### Demographic data and laboratory characteristics of dengue fever with co-infection

Data on patient age, gender, platelet count, hematocrit (Hct), and results from the rapid diagnostic test (RDT, SD BIOLINE Dengue Duo kit) were collected from 82 human cases. Of these, 62 were identified as DENV mono-infections, and 20 as co-infections. The average age of patients with DENV mono-infection was 51.05 years (range: 17-78), slightly higher than the average age of patients with co-infection, which was 47.25 years (range: 29-76). Males were more frequently represented in both the mono-infection and co-infection groups, with male-to-female ratios of 2.44 and 1.50, respectively. The average platelet count and Hct percentage were similar between the two groups. In DENV co-infection cases, the average platelet count was 112,510 cells/mL, with an Hct of 39.14%, compared to DENV mono-infection cases, which had an average platelet count of 114,000 cells/mL and an Hct of 40.19%. Primary infections were more prevalent in both groups in comparison with secondary infections, with a primary-to-secondary infection ratio of 1.21 in mono-infections and 1.5 in co-infections. No statistically significant differences were found between patient with mono-infection and co-infection group in relation to age, gender, platelet count, Hct, and primary/secondary ratio (Table 3).

**Table 3:** Demographic data and laboratory characteristic.

DENV (n = 82)	DENV mono-infection (n = 62)	DENV co-infection (n = 20)	*p*-value (<0.05)
Age (years) (Min-Max)	51.05 (17-78)	47.25 (29-76)	0.353
Gender Male/Female (M/F ratio)	44/18 (2.44)	12/8 (1.5)	0.412
Platelet count (cell/mL) (Min-Max)	114,000 (37,000-253,000)	112,510 (38,000-244,000)	0.897
Hct (%) (Min-Max)	40.19 (24 -49.4)	39.14 (31 -46.9)	0.379
Primary infection	34	12	–
Secondary infection	28	8	–
Primary/secondary ratio	1.21	1.5	0.798

### Co-circulation of all four DENV serotypes in humans and mosquitoes

**Fig 1:**
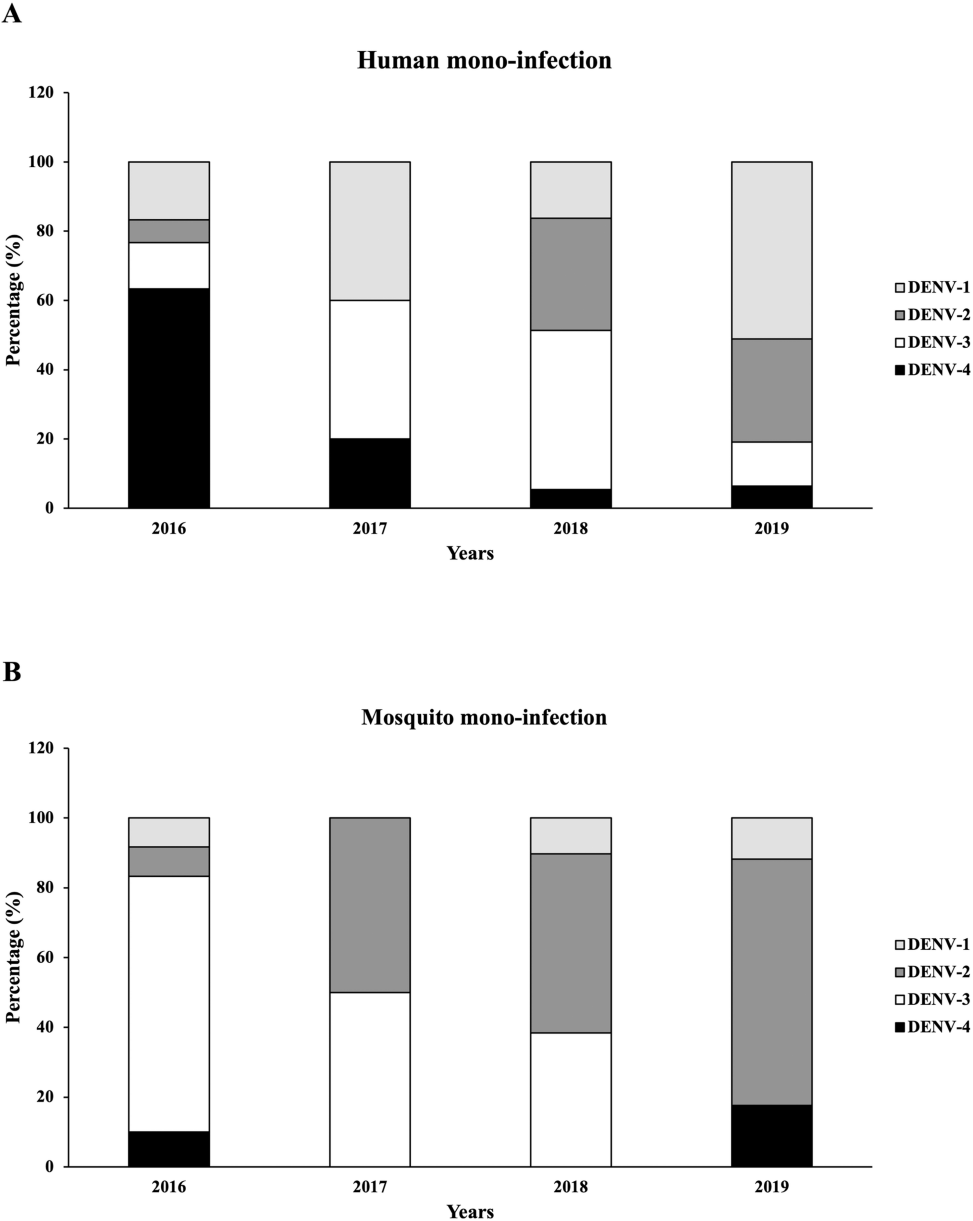
Co-circulation of DENV serotypes in human and mosquitoes from 2016-2019. Bar graphs show the number of dengue-positive cases including DENV-1, DENV-2 DENV-3 and DENV-4 in human samples (A) and mosquito samples (B).

From 390 human plasma samples collected in this study, 154 (39.49%) were successfully serotyped. Of the 1157 samples of field-caught female Aedes mosquitoes, 165 (14.26%) samples were successfully serotyped. All four serotypes of DENV were found circulating in both human and mosquito samples. The prevalence of DENV mono-infection among human DENV positive cases was 77.27% (119 cases) which was higher than that in mosquito (71.52% (118 samples)). Different predominant serotypes were found in each host and in each endemic year (Fig 1, Tables 4 and 5). In 2016, DENV-4 was dominant (63.33%) among human cases, followed by DENV-1 (16.67%), DENV-3 (13.33%) and DENV-2 (6.67%). There were low case numbers in 2017, but increased again in 2018, where DENV-3 (45.95%) was the dominant serotype followed by DENV-2 (32.43%), DENV-1 (16.22%) and DENV-4 (5.41%). In 2019, DENV-1 (51.06%) was highest followed by DENV-2 (29.79%), DENV-3 (12.77%) and DENV-4 (6.38%). In the mosquitoes in 2016, DENV-3 (73.33%) was dominant followed by DENV-4 (10%), DENV-1 (8.33%) and DENV-2 (8.33%). Similar to the human cases, there were few positive mosquitoes in 2017. The predominant serotypes in mosquitoes shifted in 2018 and 2019 with DENV-2 (51.28%), DENV-3 (38.46%), DENV-1 (10.26%) and no DENV-4 in 2018 and DENV-2 (70.59%), DENV-4 (17.65%), DENV-1 (11.76%) and no DENV-3 in 2019. Low prevalence of DENV in both humans and mosquitoes was observed in 2017 (Fig 1).

**Table 4:** Patients infected with single and multiple dengue serotypes, by year of collection.

Human	Number of patients (percentage)				
	Total	Years			
		2016	2017	2018	2019
Recruited patients	390	124	28	112	126
Serotypable	154 (39.49%)	41 (33.06%)	5 (17.86%)	50 (44.64%)	58 (46.03%)
Mono-infections	119 (77.27%)	30 (73.17%)	5 (100%)	37 (74.00%)	47 (81.03%)
DENV-1	37 (31.09%)	5 (16.67%)	2 (40%)	6 (16.22%)	24 (51.06%)
DENV-2	28 (23.53%)	2 (6.67%)	0	12 (32.43%)	14 (29.79%)
DENV-3	29 (24.37%)	4 (13.33%)	2 (40%)	17 (45.95%)	6 (12.77%)
DENV-4	25 (21.01%)	19 (63.33%)	1 (20%)	2 (5.41%)	3 (6.38%)
Co-infections	35 (22.73%)	11 (26.83%)	0	13 (26.00%)	11 (18.97%)
DENV-1,2	3 (8.57%)	0	0	0	3 (27.27%)
DENV-1,3	6 (17.14%)	0	0	3 (23.08)	3 (27.27%)
DENV-1,4	6 (17.14%)	4 (36.36%)	0	1 (7.69%)	1 (9.09%)
DENV-2,3	9 (25.71%)	0	0	6 (46.15%)	3 (27.27%)
DENV-2,4	1 (2.86%)	1 (9.09%)	0	0	0
DENV-3,4	6 (17.14%)	6 (54.55%)	0	0	0
DENV-1,2,3	1 (2.86%)	0	0	1 (7.69%)	0
DENV-1,3,4	1 (2.86%)	0	0	0	1 (9.09%)
DENV-2,3,4	0	0	0	0	0
DENV-1,2,3,4	2 (5.72%)	0	0	2 (15.38%)	0

**Table 5:** Mosquitoes infected with single and multiple dengue serotypes.

Mosquito	Number of mosquitoes (percentage)				
	Total	Years			
		2016	2017	2018	2019
Recruited mosquitos	1157	327	83	458	289
Serotypable	165 (14.26%)	73 (22.32%)	4 (4.82%)	67 (14.63%)	21 (7.27%)
Mono-infections	118 (71.52%)	60 (82.19%)	2 (50%)	39 (58.21%)	17 (80.95%)
DENV-1	11 (9.32%)	5 (8.33%)	0	4 (10.26%)	2 (11.76%)
DENV-2	38 (32.20%)	5 (8.33%)	1 (50%)	20 (51.28%)	12 (70.59%)
DENV-3	60 (50.85%)	44 (73.33%)	1 (50%)	15 (38.46%)	0
DENV-4	9 (7.63%)	6 (10%)	0	0	3 (17.65%)
Co-infections	47 (28.48%)	13 (17.81%)	2 (50%)	28 (41.79%)	4 (19.05%)
DENV-1,2	5 (10.64%)	1 (7.69%)	0	3 (10.71%)	1 (25%)
DENV-1,3	2 (4.26%)	2 (15.38%)	0	0	0
DENV-1,4	0	0	0	0	0
DENV-2,3	35 (74.47%)	8 (61.54%)	2 (100%)	22 (78.57%)	3 (75%)
DENV-2,4	0	0	0	0	0
DENV-3,4	0	0	0	0	0
DENV-1,2,3	2 (4.26%)	0	0	2 (7.14%)	0
DENV-1,3,4	0	0	0	0	0
DENV-2,3,4	3 (6.38%)	2 (15.38%)	0	1 (3.57%)	0
DENV-1,2,3,4	0	0	0	0	0

### Prevalence and characteristics of DENV co-infection in human and mosquitoes

The occurrence of multiple dengue serotype co-infections in each host were observed and reported as a prevalence (%). The prevalence of DENV co-infection in humans was 22.73% (35 out of 154 cases) and female Aedes mosquitoes 28.48% (47 out of 165 samples). The combinations included double, triple and quadruple co-infections as shown in Table 4 for human cases and Table 5 for mosquito samples. DENV double serotype co-infection was the most common pattern, with 88.56% and 89.37% in human and mosquito samples, respectively. Triple serotype co-infection was 5.72% and 10.64% in human and mosquito samples. Quadruple serotype was found only in human cases, with a frequency of 5.72%. The variety of serotype pattern in human was greater than in mosquitoes. Nine concurrent patterns were found in human; 1/2, 1/3, 1/4, 2/3, 2/4, 3/4, 1/2/3, 1/3/4 and 1/2/3/4 and five concurrent patterns were found in mosquito; 1/2, 1/3, 2/3, 1/2/3 and 2/3/4. The combinations mostly included the predominant serotype of that host in the year in question. In humans, in 2016, the co-infection patterns were 1/4 (36.36%), 2/4 (9.09%) and 3/4 (54.55%). In 2018, the most common co-infection pattern was 2/3 (46.15%). In 2019, the co-infection patterns were 1/2 (27.27%), 1/3 (27.27%) and 1/4 (9.09%). Similar results were found in mosquitoes where serotype DENV-3 was dominant in 2016 and occurred in the 2/3 (61.54%) and 1/3 (15.38%) combinations. In 2018, the co-infection patterns of DENV in mosquitoes were 2/3 (78.57%), 1/2 (10.71%), 1/2/3 (7.14%) and 2/3/4 (3.57%). There were only 4 co-infected mosquitoes in 2019 of which all were infected with DENV-2, either as 1/2 and 2/3 combinations.

### Localization of DENV co-infection in human and mosquito

**Fig 2:**
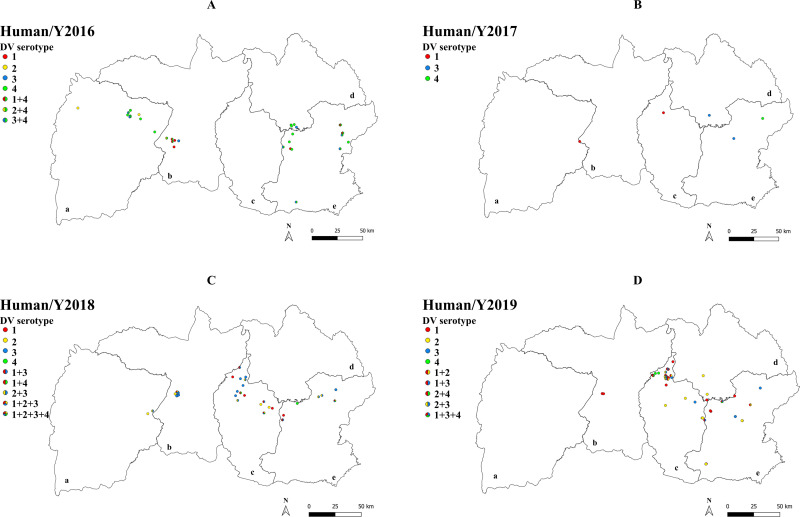
Localization of DENV mono-infection and co-infection in humans in northeastern Thailand during 2016-2019. GIS maps represent the distribution of DENV mono- and co-infection in humans in several provinces of northeastern Thailand: Chaiyaphum (a), Khon Kaen (b), Maha Sarakham (c), Kalasin (d) and Roi-Et (e).

**Fig 3:**
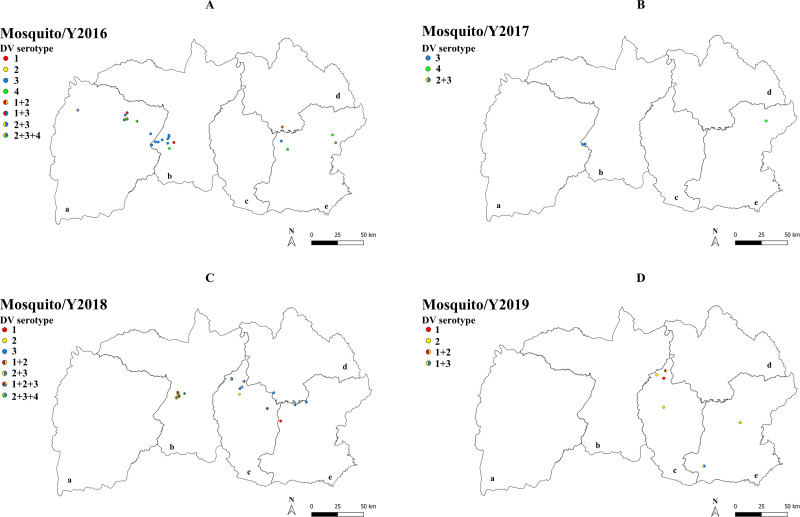
Localization of DENV mono-infection and co-infection in mosquitoes in northeastern Thailand during 2016-2019. GIS maps represented the distribution of DENV mono- and co-infection in the provinces of Chaiyaphum (a), Khon Kaen (b), Maha Sarakham (c), Kalasin (d) and Roi-Et (e).

The location of DENV serotype mono- and co-infections in humans and mosquitoes for each study year are shown in Figs 2 and 3. From latitude and longitude data collected from 353 households, dengue serotyping was positively detected on human samples from 140 households and on mosquito samples from 56 households. Although dengue in humans and mosquitoes emerged in similar geographic areas, the serotypes in mono-infections and co-infections differed between mosquitoes and humans within each year.

## Discussion

Co-circulation of all four DENV serotypes has been found in Thailand, a dengue hyperendemic country, since the first outbreak in 1958 [23]. However, co-infection with multiple DENV serotypes has rarely been described especially in humans and the mosquito vector. Mono-infections showed different dominant serotypes between humans and mosquitoes within each year. Serotype substitution in humans is thought to be influenced by preexisting immunity, whereby the immune response to a previously encountered serotype suppresses its re-infection, thereby allowing a different, non-immune or low-immunity serotype to become the cause of subsequent infection [12,13]. Our results showed different dominant serotypes in each year and in each host similar to Santos et al. [24].

This study is among the first to report DENV co-infection in field-caught mosquitoes in Thailand. The prevalence of DENV infection in mosquitoes in this study (14.26%) is slightly higher than that from the similar study by Wijesinghe et al. [25] that collected female *Aedes* mosquitoes from patients’ houses. The pattern of co-infection with different DENV serotypes exhibits distinct dynamics between mosquitoes and humans. In our study, certain serotypes in mosquitoes were also identified in humans within the same year and continuing into the subsequent year. However, due to the limited data available and other factors influencing viral infection, we cannot conclusively determine a direct relationship between the serotype distribution in mosquitoes and subsequent human infections. Further research with more comprehensive data is required to support this hypothesis. In addition, we found no significant differences in demographic data and laboratory results between patients with mono-infection and co-infection.

In this study, the proportion of humans found to be infected with DENV (39.49%) was higher than that in mosquitoes (14.26%). This is not surprising since the humans were dengue cases. The frequency of co-infections was lower in humans (22.73%) than in mosquitoes (28.48%) but the variety of co-infection in humans was higher (9 patterns) than in mosquitoes (5 patterns). This may be due to the lesser complexity of immune system, and lower immune pressure in insects compared to humans, as noted by Lin et al., who reported that sequence variation of DENV was generally lower in naturally infected mosquitoes than in human hosts [26]. Additionally, dengue infection can inhibit the expression of mosquito immune molecules that usually have antiviral functions [27]. Combinations of DENV serotypes were composed of at least one dominant serotype in that endemic year [14]. Some patterns of DENV co-infection in mosquitoes did not include any serotypes found in the same area during the detected year, suggesting diverse transmission pathways. This may be due to mosquitoes infected with multiple DENV serotypes can arise in three possible ways: mosquitoes sequentially biting humans with different single infections, mosquitoes biting humans with multiple serotype infections, or vertical transmission to adult mosquitoes from egg, larvae, and pupae with additional infections.

The competition of virus replication was previously reported by Quintero-Gil et al. DENV co-infections are not uncommon, and although this competition often results in one predominant serotype, it does not completely eliminate the other strain [28]. However, we could not investigate the differences by our method.

Our previous work in the same study area indicated that DENV transmission occurring outside the residence may play a significant role in determining the DENV serotype in mosquitoes, especially since most of the cases were children and adults who spend their daytime away from their residence, e.g., at school or worksite, where they could have been exposed to mosquitoes infected with other serotypes [19]. The DENV serotype in humans and mosquitoes in the same location were different even though the collection time was within 24 h. However, combinations of DENV serotypes contained at least one serotype that existed in the location in each host.

The clinical severity following DENV co-infection is controversial [16,17]. Our findings confirmed results from previous studies showing that the co-infection of DENV serotypes is not related to clinical severity compared to single serotype infection [17,29]. Although DENV serotype co-infection in this study was not related to severity, we cannot overlook the impact of co-infection in host immune response especially antibody production from different serotypes which may influence disease outcome, phase of endemic serotype and vaccine development. Therefore, understanding of preexisting antibodies in dengue patients with co-infections should be considered.

## Supporting information

S1 Fig Melting curve and gel electrophoresis of dengue serotyping from modified Lanciotti’s detection method. (A) Melting curve and gel electrophoresis of PCR product from 1st round for screening mosquito pool samples by D1-Fw and D1-Rv. M is 1 Kb marker. Nrx is negative control. Nmq is dengue negative mosquito control. P1 is dengue positive control. #1-#4 are dengue negative mosquito pool samples. #5-#9 are dengue positive mosquito pool samples. (B) Melting curve and gel electrophoresis of PCR product from 2^nd^ round for serotyping individual mosquito sample by D1-Fw and TS1-TS4-Rv. M is 1 Kb marker. Nrx is negative control. Nmq is negative mosquito control. P1 is positive control for each dengue serotype. #1-#4 are dengue negative mosquito samples. #5-#9 are dengue positive mosquito samples.
(TIF)

S1 Data GIS data for Figs 2 and 3. (XLSX)
