# Supplementary figures and images for: Prevalence and characteristics of dengue virus co-infection in patients and mosquitoes collected from patients’ houses
Source: PLoS One. 2025 Mar 27;20(3):e0314553. doi: 10.1371/journal.pone.0314553 (PMC11949361; doi:10.1371/journal.pone.0314553)

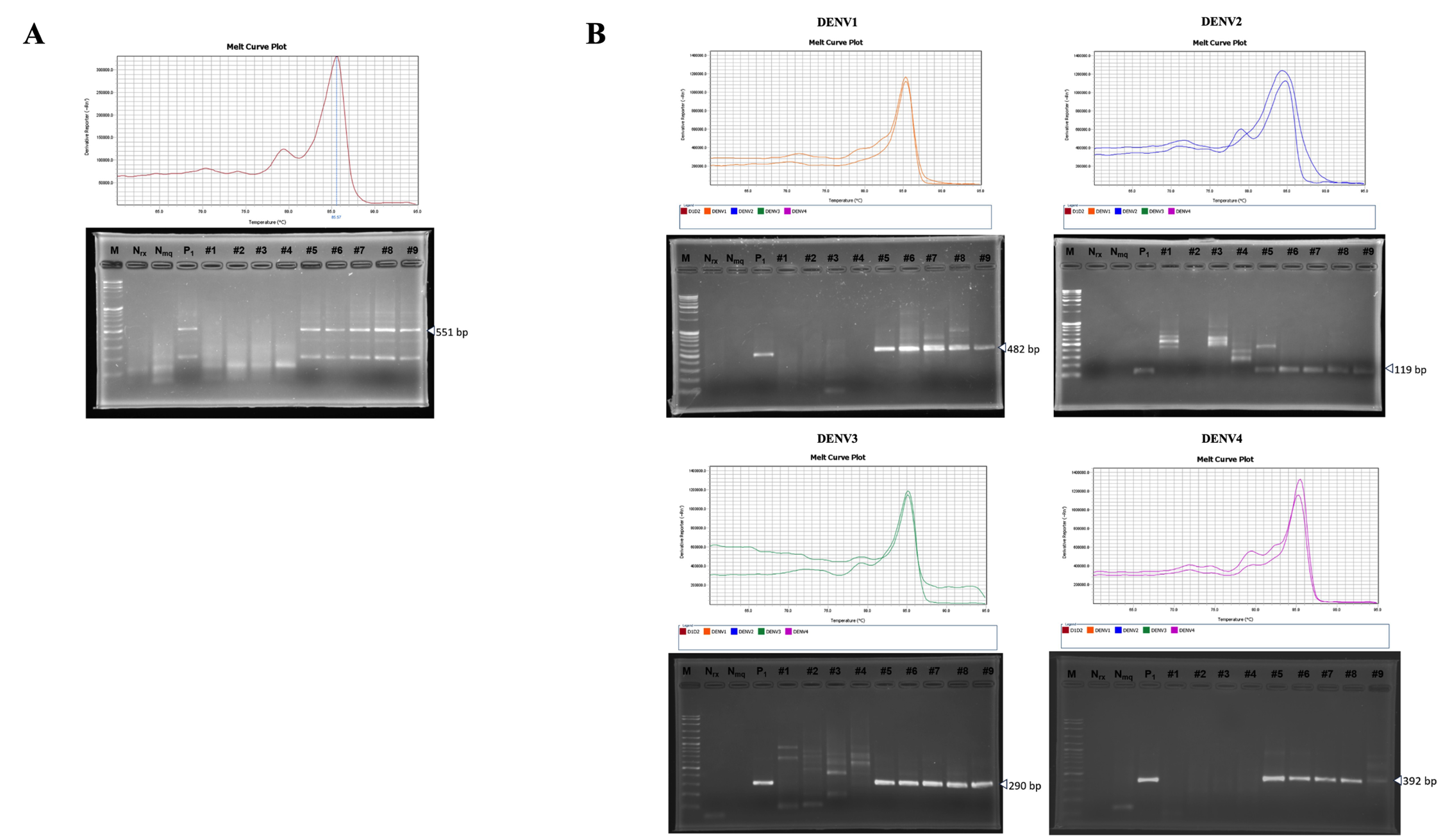

Supplement: S1 Fig — (A) Melting curve and gel electrophoresis of PCR product from 1st round for screening mosquito pool samples by D1-Fw and D1-Rv. M is 1 Kb marker. Nrx is negative control. Nmq is dengue negative mosquito control. P1 is dengue positive control. #1-#4 are dengue negative mosquito pool samples. #5-#9 are dengue positive mosquito pool samples. (B) Melting curve and gel electrophoresis of PCR product from 2nd round for serotyping individual mosquito sample by D1-Fw and TS1-TS4-Rv. M is 1 Kb marker. Nrx is negative control. Nmq is negative mosquito control. P1 is positive control for each dengue serotype. #1-#4 are dengue negative mosquito samples. #5-#9 are dengue positive mosquito samples. (TIF) [file pone.0314553.s001.tif]
